# Privacy and Ethics in Pediatric Environmental Health Research—Part II: Protecting Families and Communities

**DOI:** 10.1289/ehp.9004

**Published:** 2006-08-14

**Authors:** Celia B. Fisher

**Affiliations:** Center for Ethics Education, Fordham University, Bronx, New York, USA

**Keywords:** communities, confidentiality, culture, environmental health, ethics, families, genetic determinants, informed consent, pediatric research, prenatal testing, privacy

## Abstract

**Background:**

In pediatric environmental health research, information about family members is often directly sought or indirectly obtained in the process of identifying child risk factors and helping to tease apart and identify interactions between genetic and environmental factors. However, federal regulations governing human subjects research do not directly address ethical issues associated with protections for family members who are not identified as the primary “research participant.” Ethical concerns related to family consent and privacy become paramount as pediatric environmental health research increasingly turns to questions of gene–environment interactions.

**Objectives:**

In this article I identify issues arising from and potential solutions for the privacy and informed consent challenges of pediatric environmental health research intended to adequately protect the rights and welfare of children, family members, and communities.

**Discussion:**

I first discuss family members as secondary research participants and then the specific ethical challenges of longitudinal research on late-onset environmental effects and gene–environment interactions. I conclude with a discussion of the confidentiality and social risks of recruitment and data collection of research conducted within small or unique communities, ethnic minority populations, and low-income families.

**Conclusions:**

The responsible conduct of pediatric environmental health research must be conceptualized as a goodness of fit between the specific research context and the unique characteristics of subjects and other family stakeholders.

In pediatric environmental health research, information about family members is often directly sought or indirectly obtained in the process of identifying child risk factors and helping to tease apart and identify interactions between genetic and environmental factors. For example, research on neurodevelopmental disorders such as autism or other developmental disabilities may benefit from biological or psychological testing of healthy siblings, the collection of health or occupational information from parents, the sampling of home items for toxicants, or observation of family social interactions. A common model of survey and epidemiologic research is to ask the primary participant (e.g., the child or adolescent) for information about the home environment that may elicit personal data about family members. For example, a study of pesticide exposure may ask children or adolescents about parental occupation and hygiene habits (e.g., does the parent wear his/her work shoes into the house?). Research on the effects of secondary smoke on children’s respiratory health may include questions directed to the child regarding parent or sibling smoking behaviors and health history (asthma, lung cancer, emphysema). Although parents may have given permission for their child to be involved in the research, they may not be aware of the extent of the questions asked.

Increasingly, research involving children uses longitudinal designs to identify the developmental trajectories of environmental health problems and the single or joint health effects of heredity and prenatal and postnatal exposure to environmental agents. By its nature, such research often involves asymptomatic children who will or will not be affected by environmental agents under investigation or who will develop a disorder previously not known to be associated with an environmental agent. For example, the functional expression of prenatal or postnatal exposure to environmental neurotoxins on brain development may not appear until late childhood or early adolescence or may appear in different forms throughout the life span (Weiss 2001).

Further, a disease suspected to be a product of gene–environment interactions may occur more frequently in certain population groups, and these groups may be disproportionately exposed to stigmatization when research results are disseminated. For example, research findings suggesting that passive tobacco smoke increases complications in pediatric sickle cell patients have recently been reported in the media. Because this disease disproportionately affects African Americans, epidemiologic studies asking children about their parents’ smoking behaviors may unfairly place this population at greater risk of stigmatization regarding responsible parenting (West et al. 2003).

The direct and indirect implications of privacy violations on the health and social welfare of families and communities places the adequacy of privacy protections at the forefront of ethical concern in the design, implementation, and dissemination of research on children’s environmental diseases [National Research Council (NRC) 2005]. In this article I explore issues and potential solutions for the privacy and informed consent challenges of pediatric environmental health research to adequately protect the rights and welfare of family members and communities.

## Are Family Members Research Participants?

Institutional review boards (IRBs) reviewing studies in which children describe family behaviors may not consider parents or siblings research participants and therefore fail to consider potential privacy risks to family members in the risk–benefit analysis (Mathews 2000). Federal regulations governing protection of human subjects in research define a “human subject” as a living individual about whom an investigator obtains data through intervention or interaction with the individual or about whom the investigator has recorded individually identifiable private information. Individuals who are not considered human subjects under this definition are thus not offered protections under current federal regulations (Botkin 2001). When secondary participants are not considered human subjects, ethical procedures for obtaining informed consent and protecting confidential information may be neither required nor considered.

### Identifiable data

When there is no direct intervention or interaction with an individual, in order for a person to be considered a “human subject” under federal regulations, the individual’s identity must be readily ascertained by the investigator or associated with the information [Department of Health and Human Services (DHHS) 2005, 45 CFR 46.102(f)]. As Botkin (2001) has pointed out, whether a family member is protected under federal regulations for human subjects research thus depends on whether the data include unique individual identifiers (e.g., name, address, social security number) or information from which the family member’s identity can be easily ascertained (e.g., the child’s unique identifiers or data on individual characteristics such as sex, age, ethnicity, and education in combination with a rare environmental health problem investigated at an identified data collection site).

Family privacy concerns may not be an issue when data collected at a single point in time from a large geographically dispersed sample are anonymized and reported in the aggregate. By contrast, family privacy concerns may be raised *a*) when anonymous data are collected from a small sample of individuals with a rare environmental health risk in a small geographic area or identified health care or school setting, or *b*) when longitudinal investigations require that unique identifiers and contact information is linked to subject codes, and the primary participant shares the same address and surname as other family members.

### Genetic family privacy

Data collected on a child’s genetic code provides probabilistic health information about the child’s parents and siblings. Recognizing the risk to both the child and close relatives if private genetic information is disclosed, Annas et al. (1995) proposed that protections for individual genetic privacy must encompass not only the collection and storage of DNA samples (typically in the form of blood samples), but the information obtained from analyzing DNA samples and distribution of such samples to other investigators.

Under Annas et al.’s proposed Privacy Act of 1995, private genetic information includes

any information about an identifiable individual that has been obtained (1) from an analysis of the individual’s DNA; (2) from an analysis of the DNA of a person to whom the individual is genetically related; (3) from knowing the status of the individual in a pedigree or family history that has been developed or analyzed for a particular hereditary condition; and that (4) confirms the diagnosis of a disease; (5) determines the presence of a gene or genes, or a specific gene marker or gene markers; (6) indicates that the individual is at increased or decreased risk of developing a disease as a result of having inherited a gene; or (7) establishes that the individual is a carrier of a gene. (Annas et al. 1995)

To protect the privacy rights of family members, Annas et al. (1995) recommend that individuals who will be involved in pedigree or genetic linkage analysis be counseled on the privacy implications of this research, including the fact that other family members may find out private genetic information about them and that during the course of the study it may be determined that some family members are not in fact genetic relatives. At the same time, individuals should have access to individual records if such access does not violate the privacy rights of other family members (Annas et al. 1995). The proposed act has not been formally adopted; and although the Health Insurance Portability and Accountability Act (HIPAA) (2002) has increased protection of identifiable genetic information that is used for the assessment or treatment of an individual, it does not directly apply to privacy of genetic information obtained solely for the purpose of research.

### Privacy and harm

Classifying family members as research participants affords them certain protections under federal rules. Two of these protections are informed consent and evaluation of potential benefits and risks of research participation. If information regarding family members is considered identifiable and private, investigators may consider whether to request from their IRB a waiver of informed consent from family members. Federal regulations permit IRBs to grant a waiver of informed consent for research if it presents no more than minimal risk, the waiver would not adversely affect participants’ rights or welfare, the research could not be practicably carried out without the waiver, and, whenever appropriate, the participants are provided with additional pertinent information [DHHS 2005, 45 CFR 46.117(d)]. In requesting consent waivers for family members, investigators must be able to demonstrate to IRBs that the probability and magnitude of privacy harms that would reasonably be expected does not rise above minimal risk defined as “the probability and magnitude of harm or discomfort anticipated in the research are not greater in and of themselves than those ordinarily encountered in daily life or during the performance of routine physical or psychological examinations or tests” [DHHS 2005, 45 CFR 46.102(i)].

#### Magnitude and probability of harm

One element of the minimal risk criteria requires a decision regarding whether and to what degree research procedures may harm participants. Harm may be incurred by release of private information obtained through primary participant reports about parenting behaviors or health behaviors, or biological markers obtained from the primary participant, not otherwise available to physicians, health insurers, school officials, child welfare services, the courts, or other family members. Such harms can include social stigma, school or employment discrimination, criminal charges, or limits on health coverage. The magnitude of harm of disclosure of data collected during environmental health investigations will depend on the type of information collected. Will biological markers reveal a previously undetected probability of a genetic health disorder or presence of an environmental toxicant that will jeopardize health or home insurance? Might disclosure of children’s survey responses regarding the presence of legal or illegal toxicants in the home prompt a child neglect or criminal investigation?

The extent to which the magnitude of potential harm will curtail waiver of family member consent depends on the “probability” of harm. The probability that data collection will lead to harm depends on the type of information collected and whether sound policies for protecting confidentiality have been put into place. For example, Knoppers (2000) argues that in most instances stored genetic information does not provide sufficient information about disorders resulting from multiple allele–environment interactions to cause privacy harms. The probability of harm can also be minimized through confidentiality procedures. For example, investigators can obtain a Public Health Service (PHS) Certificate of Confidentiality to protect research records from subpoena for criminal investigations regarding family use of illicit substances. The certificate will not, however, protect against court-ordered requests for information regarding child abuse or neglect (Fisher et al. 1996).

Some assume that the use of children’s reports of parental behaviors will not meet legal or health care standards that could endanger participants. Botkin (2001) presents a cogent argument against this position, noting that because all science rests on the validity of data collected, investigators designing a study must provide ample evidence of the validity of second-hand reports if they are relying on such reports as a means of collecting data about familial variables interacting with environmental factors.

#### The ordinarily encountered standard

Whether the degree of probability and magnitude of harm merits consideration of a consent waiver depends on whether the harms are those usually encountered in daily life or during routine physical or psychological examinations. To evaluate the privacy risks under this criterion, investigators must determine whether the behavioral reports or biological tests used in the research reveal information that practitioners normally acquire during routine visits or that school staff acquire during routine child testing or parent interviews. For example, physicians routinely ask about patient smoking behaviors, but do not routinely test for the presence of certain toxicants in the blood stream. Schools may routinely conduct tests of reading skills, and parent–teacher interviews may routinely include questions about aspects of the home environment that may or may not be conducive to homework or school performance, but do not ordinarily include questions about the presence of toxic agents in the home. In addition, private information that is collected under routine medical or school procedures may enjoy greater federal protections against disclosure under HIPAA (2002, 45 CFR Part 160 and Subparts A & E of Part 164) and the Family Educational Rights and Privacy Act (1974, 20 U.S.C. 1232-34 CFR Part 99).

### Protecting family privacy rights

In designing protections for human participants, investigators must determine whether the collection of personal information about family members from the primary participant raises issues of privacy that require family members themselves to be considered research participants (Botkin 2001; NRC 2005). If it is determined that privacy concerns necessitate designation of family members as human participants, then investigators need to either address these issues in the human subjects protection procedures submitted to their IRBs (e.g., informed consent; minimizing risks of disclosure of confidential information) or modify data collection procedures to ensure privacy protections.

## Longitudinal Studies and Databases

Longitudinal designs involve multiple data collection periods sometimes initiated as early as the prenatal period and continued through adolescence or adulthood. The type of personal information collected and data collection methods typically change as the child matures. For example, biological markers may be acquired through umbilical cord blood at birth and by saliva samples, blood or MRIs during childhood and adolescence. Behavioral information may be collected from parental reports during the preschool and early school years and through self-reports during late childhood and early adolescence.

### Anonymizing longitudinal data

Use of previously collected data following participant withdrawal does not present the same ethical challenges when cross-sectional designs are used, because such designs permit anonymizing of data. By contrast, longitudinal designs must establish some way of identifying participants to link information obtained at different data collection points. Investigators can deidentify information for use by other investigators during or after the collection of data without risking the privacy rights of participants in most cases.

### The ongoing nature of consent to longitudinal studies

Given the lapses between data collection periods, the changing nature of data collection, and children’s developing ability to understand their right to privacy and confiden-tiality, the privacy rights of child participants in longitudinal studies are best protected by viewing parental permission and child assent as ongoing educational processes that are monitored and repeated at appropriate periods (Fisher et al. 1996). Reassent and parental permission procedures conducted at appropriate data collection intervals gives children and parents an opportunity to reevaluate the privacy risks of continued participation in light of the child’s increasing maturity, social experience, and evolving social, political, and economic climates. In addition, informed consent must be newly obtained for children who during the course of the study reach the legal age for consent, although under some circumstances IRBs may waive the consent requirement (DHHS 2006). The possibility that some families may drop out of the research when given this opportunity should be factored into initial estimations of sample sizes required for sufficient power.

### The right to withdraw data

The storage of biological and behavioral materials in data banks raises difficult consent challenges in almost all health research settings, but especially in environmental health research involving children. First, as noted by Chen et al. (2003), there is little scientific or public consensus on whether individuals ought to be permitted to withdraw previously collected research samples if they exert their right to withdraw from the study. This issue becomes especially challenging when children whose parents had given permission for the child’s materials to be banked reaches the legal age of consent and wish their data to be withdrawn. HIPAA regulations relevant to the use of previously collected protected health information (PHI) for research purposes may set a precedent for such consensus. HIPAA explicitly provides an exception to the right of research participants to revoke their authorization for the future use of such information, but it is less clear on the use of the samples themselves for future research. If the PHI has already been obtained and used on the basis of the original authorization, the investigator may maintain data analysis based on the information, although no additional information may be used or disclosed following revocation [DHHS 2005, 45 CFR 164.508(b)(3)(i); Fisher 2003a]. With the advances in technologies for analysis and characterization of genetic information from stored samples, this issue may become even more challenging.

### Ethical distinctions between guardian permission and child assent

At minimum, initial guardian permission and repermission procedures should include a description of the investigators’ plans for future use of already collected data in the event of participant withdrawal. This provides an opportunity for guardians who have concerns regarding future use to refuse to permit their child to participate at the onset of a longitudinal study or to dissent to continued participation as data collection procedures change over time.

Informing guardians about planned uses for stored data on participant withdrawal does not resolve privacy concerns for the children involved. First, in studies initiated prenatally or at birth, the child has no role in the participation decision. Children and young adolescents informed during reconsent procedures about plans for future use of collected data have neither the mature cognitive skills nor the experience to understand the privacy implications of such policies. In appreciation of the unique nature of children’s research, it may be ethically appropriate to permit the withdrawal of data held in data banks at the participant’s request. This policy need not extend indefinitely. Rather, the right of participants to withdraw their data might be extended only until they reach the age of majority or until the study has been completed and data anonymized. Again, investigators should take into account the possibility of such data withdrawals in their power estimates (Chen et al. 2003).

## Small-Area, Unique, and Hard-to-Reach Populations

Individuals living in small, unique areas where exposure to environmental toxicants are hypothesized to be related to high proportions of childhood disorders run greater risks of personal identification than participants from larger samples. For example, participants or their families may be personally identified when demographic information permits detailed cross-tabulation of small numbers of participants (Taylor 2001). The identification of individual health service providers may be similarly vulnerable. Do principles of justice require that environmental researchers accept responsibility for the effect of knowledge dissemination on small or unique populations?

Wing (2002) describes a poignant situation in which members of a small community participated in research designed to uncover whether noxious odors coming from a local hog production plant were indicative of dangerous levels of toxicants. The investigators made every effort to protect community confidentiality by removing demographic information on census block groups used to match communities and exact information about the size of the hog operations. Nonetheless, because the investigators were associated with a public university, the pork industry succeeded in obtaining a court order for all documentation from notes, work papers, and participant payments. As a result, community members were threatened by the hog industry employers, and some lost their jobs when they agreed to provide to researchers clinical data to test whether such health concerns were legitimate. One important lesson from this incident is that investigators should not promise confidentiality without knowing in full what they can keep confidential.

### The need for small population privacy protections

When working with rural or small and unique ethnic populations, investigators need to be aware of the added difficulties of protecting the confidentiality of individuals and their communities. When health data collection is conducted in institutional settings, investigators need to take steps to protect participant privacy, recognizing that institutional staff may have different disclosure and reporting obligations or may use the knowledge in ways that participants and their guardians may find undesirable (Fisher et al. 2002).

Conventional confidentiality procedures may not be sufficient to protect the privacy of individuals in small, rural, or closely knit ethnic neighborhoods. For example, in such settings, recruitment efforts, if they involve door-to-door sampling or walk-ins to an easily identifiable research recruitment location, can alert community members to the nature of a child’s actual or suspected disorder. Similarly, media reports of research describing the unique characteristics of unnamed, but small or unique ethnic communities or tribes may lead to public identification and stigma (Mohatt and Thomas 2006; Noe et al. 2006; Norton and Manson 1996; NRC 2005). Finally, procedures for protecting confidentiality may be seriously challenged in epidemiologic or ethnographic studies of small communities, when interviewers drawn from the community have other role relationships with the participant families (Fisher et al. 2002).

### Justice and research burdens

Federal guidelines are designed to protect individuals but not populations or communities from risk (American Academy of Pediatrics 2004). Principles of social justice make it critical for investigators to consider whether families from lower socioeconomic statuses are unfairly burdened with the confidentiality risks associated with environmental research. Poor families are more likely to live near toxic facilities or waste sites or be exposed to residential or occupational environmental inequities such as lead poisoning, air pollution, food contaminants, and industrial toxicants (Lambert et al. 2003). Although individuals with higher incomes are also exposed to these environmental hazards, from a practical perspective it may be easier to identify research populations in communities in which risk is concentrated. Environmental researchers must thus consider whether poorer populations are unjustly asked to bear the burden of privacy risks for the benefit of large-scale public health.

### Defining community

Participatory research is a promising approach to addressing potential injustices of including small or disenfranchised populations in research. A community can refer to individuals from a common ethnic group who share culture, traditions, language, and religion or to persons from different ethnic groups who share similar barriers to employment, education, housing, or quality health care rooted in exposure to historical discrimination and contemporary racial and ethnic biases (Fisher et al. 2002). Identifying persons who can best represent research-relevant concerns of prospective participants requires an understanding of the social structures and relationships that define a community (Weijer and Emanuel 2000).

The particular protections that community consultation provides will vary with the extent of community cohesion and whether there are individuals with legitimate authority and understanding who can reflect the views and interests of prospective participants. For example, families living on American Indian and Alaska Native land may agree with the tradition of empowering tribal councils with authority to make binding decisions about research participation of tribal members (Norton and Manson 1996). In contrast, children and families drawn from diverse Spanish-speaking or Asian-American groups may have different countries of family origin, immigration histories, ethnic identifications, and levels of acculturation, making it more difficult for investigators to identify appropriate cultural representatives (Fisher et al. 2002). There can also be instances where interests of the larger community are not congruent with the best interests of vulnerable children and families within the community (Macklin 1999). Parents of children with susceptibility to environmental health disorders may favor an environmental intervention in the hopes that it will prevent the onset of an environmental disease, whereas nonaffected members of the community may reject the trial out of concern about group stigma (Fisher et al. 2002).

## Conclusions

Children’s privacy rights need not be conceptualized as isolated or isolating (Walker 2002). Children’s involvement in environmental health research does not occur in a vacuum, but within the context of federal, institutional, and family protections (Fisher and Masty 2006; Kodish 2003). First, investigators with their IRBs determine whether the balance of research risks and prospective benefits are ethically justified for the population to be recruited. Second, parents decide whether the risk–benefit balance is appropriate for their own child’s unique characteristics and experiences. If parents give permission for research participation, developmentally fitted child assent procedures allow children to decide whether they wish to participate in the research procedures and purposes, as they understand them (Fisher 2003b). Creating opportunities for continued research education and periodic consent conferences can create research contexts that minimize family stress, optimize children’s input into the participation decision, and ensure that privacy needs are met. Family-focused and developmentally fitted procedures that protect family member privacy while promoting child participants’ maturing autonomy are thus essential to an informed consent ethic of respect and care.
